# Cognition, depression, fatigue, and quality of life in primary Sjögren's syndrome: correlations

**DOI:** 10.1002/brb3.586

**Published:** 2016-10-13

**Authors:** Belgin Koçer, Mehmet Engin Tezcan, Hale Zeynep Batur, Şeminur Haznedaroğlu, Berna Göker, Ceyla İrkeç, Rümeysa Çetinkaya

**Affiliations:** ^1^ Department of NeurologyGazi University School of MedicineAnkaraTurkey; ^2^ Department of RheumatologyGazi University School of MedicineAnkaraTurkey

**Keywords:** cognitive dysfunction, daily‐life activities, depression, fatigue, primer Sjögren's syndrome

## Abstract

**Objective:**

The aim of the present study was to investigate the prevalence and pattern of cognitive dysfunction observed in primary Sjögren's syndrome (PSS) and to examine the relationships between cognitive abilities, depression, fatigue, and quality of life.

**Materials and Methods:**

Thirty‐two subjects with PSS were compared with 19 healthy controls on comprehensive neuropsychological, depression, fatigue, health state, and daily‐life activities tests.

**Results:**

There was low performance in Clock Drawing, COWAT, Paced Auditory Serial Addition Test (PASAT), Colorless Word Reading (Stroop1) and Recognizing Colors (Stroop2) Patterns of STROOP test, SDLT, Auditory–Verbal Learning Test (AVLT), immediate and long‐term verbal memory, Benton Judgment of Line Orientation Test (BJLOT), and in all the patterns of RCFT in PSS patients compared to the healthy control group (*p *< .05). It was observed an increased depression frequency and fatigue severity, impairment in health condition, and a decreased quality of life in PSS cases compared to the healthy controls (*p *< .05). All the depression, fatigue severity, and quality of life tests showed a significant positive correlation with each other (*p *< .05). A significant negative correlation between Clock Drawing and SF‐36‐BP (*p *= .031, *r *= −.382) and SF‐36‐GH (*p *= .027, *r *= −.392) was observed.

**Conclusions:**

Clock Drawing, PASAT, and AVLT are very useful tests to determine the subclinical and clinical cognitive dysfunction to evaluate attention, information processing speed, executive functions, and short‐term and long‐term verbal memory in PSS patients. Depression and fatigue may not affect the neuropsychological tests performance.

## 1 Introduction

Primary Sjögren's syndrome (PSS) is primarily a chronic autoimmune disease that involves lacrimal glands and salivary glands and is clinically characterized by dryness of mouth (xerostomia) and dryness of the eye (xerophthalmia). Sjögren's syndrome (SS) may develop primarily or secondary to another connective tissue disease (SLE, rheumatoid arthritis, scleroderma) (Delalande et al., 2004; Mellgren, Göransson, & Omdal, 2007). It is seen nine times more in women in the age group of 40–50 years compared to men (Morreale et al., 2014). Incidence of neurological involvement in SS is 25–30%. Involvement of the central nervous system can be seen in optic neuropathy, multiple cranial neuropathy, transverse myelitis, aseptic meningitis, encephalomyelitis, epilepsy or ischemic stroke clinic (Delalande et al., 2004; Mellgren et al., 2007). In case of xerostomia and xerophthalmia, fatigue, cognitive symptoms, and pain are frequently present (Lafitte et al., 2001).

In a limited number of studies conducted regarding PSS, cognitive dysfunction is defined with incidence rate of 44.4–50% (Harboe et al., 2009; Morreale et al., 2014). Retardation in attention, information processing speed (working memory), executive functions, verbal and visual memory, and disruption in visual–spatial perception, motor reaction time, and increase in depression and fatigue frequency are shown (Lafitte et al., 2001; Martinez et al., 2010; Mataro et al., 2003; Morreale et al., 2014; Segal et al., 2012). Mechanism of cognition disorder is explained by immune‐mediated inflammatory small‐vessel disease or direct infiltration of the brain tissue with chronic inflammatory cells (Mataro et al., 2003). Higher cognitive function disorders in the cases with anti‐SSA antibody positivity support the role of immune mechanism (Chang, Shiau, Wang, Ho, & Kao, 2002; Harboe et al., 2009; Morreale et al., 2014; Yoshikawa et al., 2012). Additionally, it is observed that depression and fatigue severity increase (Lafitte et al., 2001; Martinez et al., 2010; Segal et al., 2012; Strömbeck, Ekdahl, Manthorpe, & Jacobsson, 2003) but quality of life impairs (Baturone et al., 2009; Champey et al., 2006; Ibn Yacoub, Rostom, Laatiris, & Hajjaj‐Hassouni, 2012; Lendrem et al., 2014; Meijer et al., 2009; Segal et al., 2009) in cases with PSS compared to healthy controls.

The purpose of this study is to determine the presence, frequency, and type of cognitive dysfunction in the patients who had no symptoms and findings of other central nervous system but were diagnosed with PSS along with and without cognitive symptoms and findings. Depression, fatigue severity, health state, and daily living activities are measured in the patients by using objective tests. The correlation between presence of cognitive dysfunction and depression, fatigue, health state, and daily living activities was examined in the patients.

## 2 Materials and Methods

### 2.1 Patients

Thirty‐two patients with PSS, who were admitted to Gazi University Medical Faculty outpatient clinic of Neurology Department between July 2011 and August 2013, were included in the study. All patients met the criteria of European‐American Consensus Group for classification as PSS (Vitali et al., 2002). Twenty normal subjects were included as controls. The patients and healthy controls were matched in terms of age, sex, and education level, and there was no significant difference between the two groups. No patients or controls were taking benzodiazepines, neuroleptics, antidepressants, or alcohol, and none had a history of head injury or other central nervous system, any other connective tissue disease, and psychiatric, metabolic, or endocrinological disease (Lafitte et al., 2001; Segal et al., 2012). No history of neurological involvement of PSS was investigated. The PSS patients underwent complete physical and neurological examinations, hemogram, total biochemical and urine analyses, and complete immunological investigations, including erythrocyte sedimentation rate, antinuclear antibodies, antibodies to Ro/SSA, La/SSB, rheumatoid factor (RF), C‐reactive protein, serum IgG, IgA, and IgM, complement C3 and C4 fractions, cryoglobulinemia. Specimens of minor salivary gland biopsy were obtained from all patients according to standard procedures.

Candidates with severe motor or visual impairments that might interfere with cognitive testing were excluded from the study. All the patients had normal hearing thresholds. All the participants gave the informed consent before they were included in the study. Table 1 shows the demographic characteristics of PSS and control groups.

**Table 1:** Demographic characteristics of PSS and healthy control groups

	Patient (*n* = 32)	Control (*n *= 19)	*p*
Age	45.19±11.52	44.53±5.45	.783
Educational level (years)	9.75±3.70	11.05±3.66	.228
Duration of disease (months)	33.23±40.49		

### 2.2 Neuropsychological assessment

Before the psychometric test battery, language function was assessed and no participant showed abnormalities in either oral or written comprehension. A comprehensive neuropsychological battery of tests was used to assess the cognitive performance of PSS patients and healthy subjects. All the tests used are standard normalized tests for which normal subject performances are known. Individual test performance was considered as abnormal when it was below the normal control mean. A defect in one of the tests was interpreted as a loss of function in that area. It was considered that neuropsychological assessment revealed CNS pathological involvement when at least two test results were abnormal.

In these cases attention, information processing speed, short‐ and long‐term verbal and visual memory, and visual–spatial perception were examined. For this purpose, Clock Drawing Test, Controlled Oral Word Association Test (COWAT), Stroop (Stroop Interference Test), PASAT, Boston Naming Test (BNT), Serial Digit Learning Test (SDLT), Auditory–Verbal Learning Test (AVLT), Benton Judgment of Line Orientation Test (BJLOT), and Rey Complex Figure Test (RCFT) were administered. Attention and executive functions using Stroop test, verbal fluency using COWAT, naming confrontation using BNT, verbal learning using SDLT, and immediate, short‐ and long‐term verbal memory using RCFT were investigated and measured (Lafitte et al., 2001; Martinez et al., 2010; Segal et al., 2012; Tombaugh, Kozak, & Rees, 1999).

### 2.3 Mini‐Mental State Score

The “mini‐mental state” (MMS) is scored form of the cognitive mental status examination (Folstein, Folstein, & McHugh, 1975), which includes 11 questions, requires only 5–10 min to administer, and is therefore practical to use serially and routinely. It concentrates only on the cognitive aspects of mental functions, and excludes questions concerning mood, abnormal mental experiences, and the form of thinking. The MMSE is a paper‐based test with a maximum score of 30, with lower scores indicating more severe cognitive problems. The cut point established for the MMSE defines “normal” cognitive function and is usually set at 24, although theoretically it could fall anywhere from 1 to 30.

### 2.4 Tests for evaluating verbal memory

1. Serial Digit Learning Test: This test measures overall learning ability and short‐term memory. The test is sensitive to damage in the mesial temporal lobe, hippocampus, and other limbic system structures (Benton, de Hamsher, Varney, & Spreen, 1983; Delalande et al., 2004).
2. Boston Naming Test: This test evaluates the ability to naming and recognizing objects (Bush, Frazier, Haggerty, & Kubu, 2005).
3. Auditory–Verbal Learning Test: This test measures verbal learning and memory (Hohol et al., 1997), including functions of the left temporal zone such as immediate recall and learning of verbal information, keeping the information in memory, and storing it in long‐term memory. The test is sensitive to damage in the hippocampus and temporal lobe, particularly lesions of the left hemisphere (Grammaldo et al., 2006). The total number of words recalled during five consecutive presentations of a list of 15 words, and the number of words recalled after a 20‐min delay were recorded (Mataro et al., 2003).

### 2.5 Tests for evaluating visual memory and visual–spatial perception

1. Benton Judgment of Line Orientation Test: This test measures visuospatial perception and orientation. It is particularly sensitive to damage in the right cerebral hemisphere and right parietal lobe (Karakaş et al., 2004).
2. Rey Complex Figure Test: This is a test of visuospatial learning and delayed recall (Hohol et al., 1997). It evaluates visual perception, perceptional organization, visual learning, and storing of visual information in short‐ and long‐term memory. The test measures right hemisphere function (Karakaş et al., 2004).
3. Writing test: This test measures motor skills and writing speed (Karakaş et al., 2004).
4. 10‐Point Clock Drawing Test (CDT): The clock drawing test is a widely used test for screening cognitive impairment. It evaluates visuo‐constructive and visuospatial skills, symbolic and conceptual representation, hemiattention, semantic memory, executive function including organization, planning, and parallel processing (Ricci et al., 2016; Shulman, 2000).

### 2.6 Tests for evaluating attention and information processing speed

1. Controlled Oral Word Association Test (COWAT): This test measures verbal fluency and semantic retrieval. It assesses recall of facts and knowledge about the world (Lafitte et al., 2001; Vitali et al., 2002). The subject is requested to find words and animal names beginning with the letters K, F, A, and S, excluding proper nouns, numbers, and plurals, within 1 min (Karakaş et al., 2004).
2. Stroop Interference Test (SIT): This test measures resistance to interference, ability to perform an unusual behavior, and focused attention. It is particularly sensitive to damage in the left frontal lobe and orbitofrontal cortex (Karakaş et al., 2004).
3. Paced Auditory Serial Addition Test (PASAT): This test measures sustained attention and information processing speed (Hohol et al., 1997).

### 2.7 Assessment of depression, fatigue, health state, and daily living activities

The presence of depression was examined by using Hamilton Depression Scale and Beck Depression Inventory (BDI).

#### 2.7.1 Hamilton Depression Scale

This is a 17‐item questionnaire that measures mood. A score of ≥8 indicates clinical depression. Scores in the range of 0–7 reflect no depression and scores between 8 and 15 reflect mild depression, whereas score ≥16 is suggestive of moderate to severe depression (Hamilton, 1967).

#### 2.7.2 Beck Depression Inventory

The BDI is the most used self‐rating scales for measuring depression. BDI‐II is a 21‐item self‐report depression screening measure (Shulman, 2000). Each item is rated on a 4‐point Likert‐type scale ranging from 0 to 3, with higher scores indicating higher levels of depression. The measures ask respondents to endorse statements characterizing how they have been feeling throughout the past 2 weeks. The maximum total score for all 21 items is 63 (Segal, Coolidge, Cahill, & O'Riley, 2008). The cut‐off score of ≥13 is commonly used to identify current clinical depression (Beck, Steer, & Brown, 1996). According to the BDI‐II manual, scores of 0–13 denote minimal depression, scores of 14–19 denote mild depression, scores of 20–28 denote moderate depression, and scores of 29–63 denote severe depression (Segal et al., 2008).

The Short‐Form 36 (SF‐36) evaluating the daily living activities, EQ‐5D questionnaire evaluating the health state, and fatigue severity scale (FSS) measuring the severity of fatigue were administered.

#### 2.7.3 The medical outcomes study, 36‐item short‐form health survey (SF‐36)

The SF‐36 questionnaire is designed to evaluate health‐related quality of life within the previous 4 weeks. It involves 36 questions, with eight scales assessing two dimensions. The first dimension is physical health function and includes the following four specific scores: physical functioning (the extent to which health interferes with various activities), physical role functioning (the extent to which health interferes with usual daily activities such as work, housework, and school), bodily pain, and general health. These physical scores are summarized by the physical composite score (PCS). The second dimension is mental health function, which includes the following four specific scores: vitality, social functioning, emotional role functioning (limitations due to emotional problems), and mental health. These four mental scores are summarized by the mental composite score (MCS). Each scale gives a standardized raw score that ranges from 0 to 100, with 0 implying the worst possible health state and 100 implying the best possible health state (Ware & Gandek, 1998; Ware & Sherbourne, 1992).

#### 2.7.4 EQ‐5D1 and EQ‐5D2

Euro‐QoL‐5 dimension (EQ‐5D) is a standardized preference‐based tool for the measurement of health‐related quality of life (Mataro et al., 2003; Segal et al., 2009). EQ‐5D1 and EQ‐5D2 were performed. An individual's perception of his or her health is recorded on a 0–100 visual analog scale (VAS) with 0 being “worst imaginable health state” and 100 being “best imaginable health state” with EQ‐5D1. The EQ‐5D2 assesses five different dimensions of health (mobility, self‐care, ability to usual activities, pain/discomfort, and anxiety/depression). Each dimension can be scored by three possible responses: no problem, some/moderate problems, or severe problems, corresponding to a score of 0–2 (Segal et al., 2009). The best state was evaluated as three points and the worst state was evaluated as 15 points (The EuroQol Group, 1990).

#### 2.7.5 Fatigue severity scale

Fatigue was evaluated by using the FSS. Fatigue severity scale is a self‐administered instrument developed to assess the impact and severity of fatigue. The questionnaire includes nine statements to explore a person's severity fatigue symptoms as it relates to daily activities such as physical functioning, exercise, work, and family and social life. The scores for each item range from 1 to 7, with the lower score indicating less fatigue. In FSS, behavioral result of fatigue is evaluated by a 9‐point question. In every question, the score range is between 1 and 7 (best condition is 9 points; worst condition is 63 points). We used a cut‐off score ≥4 to define fatigue. A FSS score ≥4 reliably differentiates subjects with fatigue from control subjects (Epstein et al., 2014; Krupp, LaRocca, Muir‐Nash, & Steinberg, 1989; Segal et al., 2008).

All subjects gave their informed consent prior to sample acquisition. The study was approved by the local Ethics Committee of Gazi University School of Medicine.

### 2.8 Statistical analysis

Statistical analyses were carried out by IBM SPSS for Windows 22.0 package program. Numerical variables were summarized by the mean ± standard deviation and median [min–max] values. Qualitative variables were shown by numbers and percentages. The presence of a difference between independent groups in terms of the qualitative variables was examined by using chi‐square test or Fisher's exact test. Before the numerical variables were compared, it was checked whether the parametric test assumptions were met or not. While conformity to normal distribution was evaluated by using Shapiro–Wilk test, the homogeneity of the variances was evaluated by using the Levene's test. The presence of a difference between the independent groups in terms of numerical variables was investigated by using the independent samples *t*‐test in case that the parametric test assumptions were met and by using Mann–Whitney *U* test in case that the said assumptions were not met. The presence of a correlation between the numerical variables was determined by Spearman's correlation coefficient. Significance level was accepted as *p *< .05.

## 3 Results

### 3.1 Patients

Thirty‐two female cases diagnosed with PSS diagnosis and 19 female cases as healthy control group were included in the study. Ages were between 21 and 64 years (45.19 ± 11.52) in the PSS group and between 35 and 57 years (44.53 ± 5.45) in the control group (*p *= .783). In the PSS group, the duration of disease was between 2 months and 15 years (33.23 ± 40.49). The educational level of both groups was not different (*p *> .05) and while the educational level was (9.75 ± 3.70) years in the PSS group, it was (11.05 ± 3.66) years in the healthy control group (*p *= .228). In the PSS group, 14 cases had an education for 5–8 years, 8 cases for 9–11 years, and 10 cases for 12–15 years; and in the healthy control group, 5 cases had an education for 5–8 years, 4 cases for 9–11 years, and 10 cases for 12–15 years. Table 1 shows the demographic characteristics of PSS and healthy control groups (Table 1).

### 3.2 The findings of neuropsychological cognitive tests

**Figure 1:**
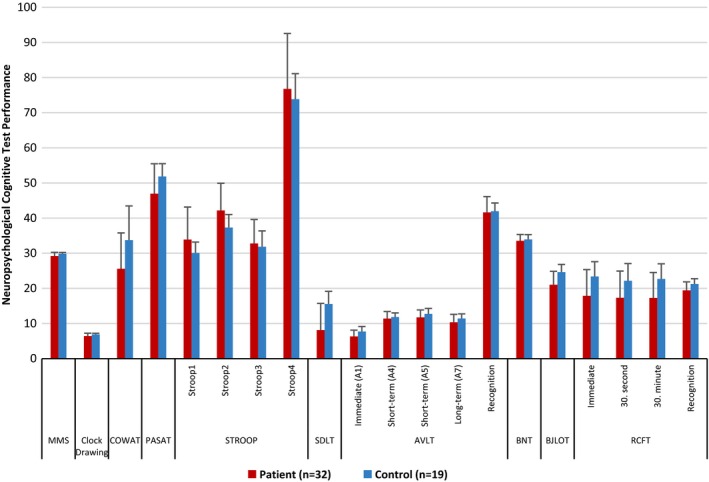
Comparison of neuropsychological cognitive test performances in PSS and healthy control groups

When the neuropsychological test performances were compared in PSS and healthy control groups, the MMS values were identified as 26–30 (29.22 ± 1.039) in patient group and as 29–30 (29.84 ± 0.375) in control group (*p *= .021). There was low performance in Clock Drawing, COWAT, PASAT, Colorless Word Reading (Stroop1) and Recognizing Colors (Stroop2) Patterns of STROOP test, SDLT, AVLT immediate verbal memory (A1) and long‐term verbal memory (A7) patterns, BJLOT, and in all the patterns of RCFT in PSS patients compared to the healthy control group (*p *< .05). There was no performance difference between the groups in the STROOP test colored word reading (Stroop3) and telling color of colored word (Stroop4) patterns, AVLT A4, A5 (short‐term verbal memory) and recognition patterns and in the BNT tests (*p *> .05) (Table 2) (Figure 1). Thus, it was observed that there was a dysfunction in the tests evaluating attention, information processing pace, verbal learning and immediate short‐term and long‐term verbal memory, and visual–spatial perception.

**Table 2:** Values of neuropsychological test performances in PSS and healthy control groups

	Patient (*n *= 32)	Control (*n *= 19)	*p*
MMSS	29.22±1.04	29.84±0.38	**.021**
Clock Drawing	6.44±0.80	6.89±0.32	**.029**
COWAT	25.59±10.19	33.74±9.72	**.007**
PASAT	46.97±8.49	51.84±3.64	**.007**
Stroop (second)			
Stroop1	33.91±9.23	30.09±3.09	**.038**
Stroop2	42.19±7.72	37.33±3.68	**.004**
Stroop3	32.80±6.79	31.86±4.50	.594
Stroop4	76.79±15.76	73.84±7.26	.368
SDLT	8.13±7.59	15.58±3.564	**.000**
AVLT			
Immediate (A1)	6.31±1.80	7.74±1.41	**.003**
Short term (A4)	11.41±2.00	11.84±1.17	.330
Short term (A5)	11.75±2.10	12.74±1.56	.081
Long term (A7)	10.34±2.28	11.42±1.31	**.037**
Recognition	41.63±4.49	41.95±2.35	.738
BNT	33.53±1.80	33.95±1.35	.387
BJLOT	21.03±3.82	24.63±2.19	**.000**
RCFT			
Immediate	17.88±7.45	23.37±4.21	**.001**
30 seconds	17.34±7.58	22.16±4.89	**.008**
30 minutes	17.28±7.23	22.68±4.30	**.002**
Recognition	19.42±2.42	21.26±1.49	**.004**

MMSS: Mini‐mental state score, COWAT: Controlled Oral Word Association Test, PASAT: Paced Auditory Serial Addition Test, SIT: Stroop Interference Test; *colourless word reading (Stroop1), recognizing colours (Stroop2), coloured word reading (Stroop3), telling colour of coloured word (Stroop4) patterns of SIT,* SDLT; Serial digit learning test, AVLT; Auditory‐verbal learning test, BNT; Boston naming test, BJLOT; Benton Judgement of Line Orientation Test, RCFT; Rey Complex Figure Test

It was observed that as the educational level increased, performance increase was found in all tests (*p *< .05). When the educational level was classified as 5–8 years, 9–11 years, and 12 years and above in both groups, it was found that the educational level of 5–8 years had performance decrease in SDLT (*p *= .034), RCFT immediate (*p *= .034), after 30 second (*p *= .026) and after 30 minute (*p *= .034) patterns, and SF‐36‐GH (*p *= .002) in the PSS group compared to the healthy control group; the educational level of 9–11 years had performance decrease in SDLT (*p *= .028), AVLT immediate verbal memory (A1) (*p *= .016) tests, BDI (*p *= .48), and SF‐36‐RE (*p *= .028) in the PSS group compared to the healthy control group; and also the educational level of 12 years and above had performance decrease in RCFT recognition (*p *= .006) pattern, EQ‐5D1 (*p *= .004), EQ‐D2 (*p *= .011), FSS (*p *= .000), SF‐36‐RP (*p *= .019), SF‐36‐BP (*p *= .023), SF‐36‐GH (*p *= .015), and SF‐36‐VT (*p *= .029) in the PSS group compared to the healthy control group.

### 3.3 The findings of depression, fatigue, health state, and daily living activity following

It was observed that there were an increase in depression frequency and fatigue severity, impairment in health condition, and a decrease in daily living activities in PSS cases compared to the healthy controls (*p *< .05). It was observed that while in PSS group there was a significant impairment in role functioning physical, bodily pain, general health, vitality, and role functioning emotional patterns of daily living activities; physical function, social functioning, and mental health did not impair (*p *> .05) (Table 3).

**Table 3:** Comparison of Hamilton Depression Scale, Beck Depression Inventory, FSS, EQ‐5D1, EQ‐5D2, and Short‐Form‐36 in PSS and healthy control groups

	Patients (*n *= 32)	Control (*n *= 19)	*p*
HDS	6.69±5.47	3.00±2.65	**.010**
BDI	11.63±6.60	6.26±3.51	**.002**
EQ‐5D1	65.16±15.42	80.00±14.81	**.000**
EQ‐5D2	7.56±1.90	5.95±1.22	**.002**
FSS	28.34±14.94	17.47±11.40	**.009**
SF‐36			
Physical function	73.28±19.99	81.05±19.97	.108
Role functioning physical	57.81±43.27	93.42±11.31	**.006**
Bodily pain	53.44±20.26	73.16±20.56	**.001**
General health	47.53±23.14	74.76±12.82	**.000**
Vitality	32.19±19.63	50.53±17.15	**.001**
Social functioning	66.02±34.23	81.58±20.57	.145
Role functioning emotional	59.34±32.50	85.95±25.64	**.002**
Mental health	36.00±18.00	40.42±13.46	.305

HDS: Hamilton Depression Scale, BDI: Beck Depression Inventory, EQ‐5D1: Euro‐QoL‐5 dimension1, EQ‐5D2: Euro‐QoL‐5 dimension2, FSS: Fatigue Severity Scale,SF‐36: The Medical Outcomes Study, 36‐item Short Form Health Survey

While depression was regarded as available in cases obtaining eight scores and above from Hamilton Depression Scale (HDS), it was regarded as unavailable in cases with 0–7 scores. In this case, the presence of depression was determined in 40.6% (*n *= 13) frequency in PSS patients group. On the other hand, the presence of depression was determined only as 5.3% (*n *= 1) in the healthy control group.

### 3.4 Correlation between neuropsychological cognitive tests and depression, fatigue, health state, and daily‐life activities

When it was evaluated whether or not there was a correlation between the neuropsychological tests and depression, fatigue severity, health state, and daily‐life activities in PSS cases, it was observed that there was a significant negative correlation between Clock Drawing and SF‐36‐BP (*p *= .031, *r *= −.382) and SF‐36‐GH (*p *= .027, *r *= −.392). All the depression, fatigue severity, health state, and quality of life tests showed a significant positive correlation with each other (*p *< .05) (Table 4).

**Table 4:** Correlation between neuropsychological cognitive tests and depression, fatigue, health, daily‐life activities in patients with PSS

	HDS	BDI	EQ‐5D1	EQ‐5D2	FSS	SF‐36‐PF	SF‐36‐RP	SF‐36‐BP	SF‐36‐GH	SF‐36‐VT	SF‐36‐SF	SF‐36‐RE	SF‐36‐MH
MMSS													
Clock Drawing								*r *= −.382, *p *= .031	*r *= −.392, *p *= .027				
COWAT													
PASAT													
Stroop													
Stroop1													
Stroop2													
Stroop3													
Stroop4													
SDLT													
AVLT													
Immediate (A1)													
Short term (A4)													
Short term (A5)													
Long term (A7)													
Recognition													
BNT													
BJLOT													
RCFT													
Immediate													
30 seconds													
30 minutes													
Recognition													
HDS		*r *= .866, *p *= .000	*r *= −.572, *p *= .001	*r *= .662, *p *= .000	*r *= .597, *p *= −.000	*r *= −.404, *p *= .022	*r *= −.640, *p *= .000	*r *= −.455, *p *= .009	*r *= −.399, *p *= .024	*r *= −.512, *p *= .003	*r *= −.547, *p *= .001		
BDI	*r *= .866, *p *= .000		*r *= −.666, *p *= .000	*r *= .675, *p *= .000	*r *= .690, *p *= .000	*r *= −.410, *p *= .020	*r *= −.620, *p *= .000	*r *= −.501, *p *= .004	*r *= −.526, *p *= .002	*r *= −.561, *p *= .001	*r *= −.582, *p *= .000		
EQ‐5D1	*r *= −.572, *p *= .001	*r *= −.666, *p *= .000		*r *= −.715, *p *= .000	*r *= −.433, *p *= .013	*r *= .443, *p *= .011	*r *= .615, *p *= .000	*r *= .626, *p *= .000	*r *= .628, *p *= .000		*r *= .390, *p *= .027		*r *= .358, *p *= .044
EQ‐5D2	*r *= .662, *p *= .000	*r *= .675, *p *= .000	*r *= −.715, *p *= .000		*r *= .572, *p *= .001	*r *= −.488, *p *= .005	*r *= −.672, *p *= .000	*r *= −.699, *p *= .000	*r *= −.776, *p *= .000	*r *= −.480, *p *= .005	*r *= −.545, *p *= .001		*r *= −.369, *p *= .037
FSS	*r *= .597, *p *= −.000	*r *= .690, *p *= .000	*r *= −.433, *p *= .013	*r *= .572, *p *= .001		*r *= −.361, *p *= .043	*r *= −.688, *p *= .000	*r *= −.375, *p *= .034	*r *= −.449, *p *= .010	*r *= −.595, *p *= .000	*r *= −.488, *p *= .000		
SF‐36‐PF	*r *= ‐.404, *p *= .022	*r *= −.410, *p *= .020	*r *= .443, *p *= .011	*r *= −.488, *p *= .005	*r *= −.361, *p *= .043			*r *= .505, *p *= .003					
SF‐36‐RP	*r *= −.640, *p *= .000	*r *= −.620, *p *= .000	*r *= .615, *p *= .000	*r *= −.672, *p *= .000	*r *= −.688, *p *= .000			*r *= .478, *p *= .006	*r *= .587, *p *= .000	*r *= .522, *p *= .002	*r *= −.527, *p *= .002		
SF‐36‐BP	*r *= −.455, *p *= .009	*r *= −.501, *p *= .004	*r *= .626, *p *= .000	*r *= −.699, *p *= .000	*r *= −.375, *p *= .034	*r *= .505, *p *= .003	*r *= .478, *p *= .006		*r *= .703, *p *= .000	*r *= .428, *p *= .015	*r *= .678, *p *= .000		*r *= .492, *p *= .004
SF‐36‐GH	*r *= −.399, *p *= .024	*r *= −.526, *p *= .002	*r *= .628, *p *= .000	*r *= −.776, *p *= .000	*r *= −.449, *p *= .010		*r *= .587, *p *= .000	*r *= .703, *p *= .000		*r *= .465, *p *= .007	*r *= .646, *p *= .000		*r *= .458, *p *= .008
SF‐36‐VT	*r *= −.512, p = .003	*r *= −.561, *p *= .001		*r *= −.480, *p *= .005	*r *= −.595, *p *= .000		*r *= .522, *p *= .002	*r *= .428, *p *= .015	*r *= .465, *p *= .007		*r *= .483, *p *= .005		
SF‐36‐SF	*r *= −.547, *p *= .001	*r *= −.582, *p *= .000	*r *= .390, *p *= .027	*r *= −.545, *p *= .001	*r *= −.488, *p *= .005		*r *= .527, *p *= .002	*r *= .678, *p *= .000	*r *= .646, *p *= .000	*r *= .483, *p *= .005		*r *= .439, *p *= .012	*r *= .391, *p *= .027
SF‐36‐RE											*r *= .439, *p *= .012		
SF‐36‐MH			*r *= .358, *p *= .044	*r *= −.369, *p *= .037				*r *= .492, *p *= .004	*r *= .458, *p *= .008		*r *= .391, *p *= .027		

SF‐36‐PF: Short Form‐36‐Physical function, SF‐36‐RP: Short Form‐36‐Role‐functioning physical, SF‐36‐BP: Short Form‐36‐ Bodily‐pain, SF‐36‐GH: Short Form‐36‐General health, SF‐36‐VT: Short Form‐36‐Vitality, SF‐36‐SF: Short Form‐36‐Social functioning, SF‐36‐RE: Short Form‐36‐Role functioning emotional, SF‐36‐MH: Short Form‐36‐Mental health

## 4 Discussion

Cognitive dysfunction was described in patients with PSS. It was identified that there was a dysfunction at attention, information processing speed, executive functions, verbal memory and visual memory, visual–spatial perception, and lower scores on motor reaction time in the patients with PSS (Lafitte et al., 2001; Martinez et al., 2010; Mataro et al., 2003; Segal et al., 2012). In 39 PSS case series of Segal et al., the subjective cognitive symptoms, depression, fatigue severity, and pain scores were higher than the healthy control group. In the Serial Digit Learning Test that measured attention and psychomotor information processing speed, there was a significant difference in the patient group compared to the control group; the patient group had a lower performance in the STROOP test evaluating attention and executive functions, COWAT evaluating verbal fluency, HVLT‐R tests showing verbal learning and memory performance compared to control group even though it did not have a statistical significance. Mild cognitive disorder was 30% in the PSS group and 18% in the control group (*p *= .317) (Segal et al., 2012). Martinez et al. (2010) revealed a decrease at motor rate showing fronto‐subcortical dysfunction at SRT, CPT, STROOP, and WCST tests showing loss of attention, executive functions, and at test performances showing visual–spatial perception loss in 20 SS cases compared to healthy controls. In 39 PSS case series of Mataro et al. (2003), it was found that 47% of the cases had dysfunction at three or more neuropsychological tests. While in the present study a cognitive dysfunction was observed in similar cognitive areas as attention, information processing speed, executive functions, verbal memory, visual memory, and visual–spatial perception in patients with PSS; however, we used comprehensive detailed neuropsychological tests in all patients. Unlike previous studies, PASAT, BNT, all the patterns of AVLT and RCFT have applied in our study, and we found low performance in all these tests in PSS patients group compared to the healthy control group (*p *< .05). PASAT and AVLT are very useful tests to perform to the patients in a short time and to determine the subclinical and clinical cognitive dysfunction in attention, information processing speed, executive functions, and short‐term and long‐term verbal memory.

The cognitive function disorders defined within PSS are explained by frontosubcortical dysfunction. The cranial MRI abnormalities which were observed most frequently at SS were the multifocal subcortical and periventricular white matter hyperintensities in the T2 and FLAIR sections, enlargement at the sulci, and ventricular dilatation. A positive correlation was found between burden of white matter abnormalities determined in cranial MRI and retardation at the psychomotor rate, fatigue, and the increased ventricular volume and attention (Coates et al., 1999; Mataro et al., 2003). The examination of Tc‐ECD brain SPECT is sensitive to displaying subcortical cognitive dysfunction in PSS (Gerraty, McKelvie, & Byrne, 1993; Le Guern et al., 2010). In PSS cases without neurological involvement, it was found that there was a significant hypoperfusion at left frontal, left parietal, left temporal, right frontal, and right hippocampal cortex, and also it was shown that there was a strong correlation between the hypoperfusion specified at frontal, parietal, temporal, cingulate, and hippocampal areas, and the dysfunction of the executive functions (Le Guern et al., 2010). When the brain MRI was normal, it was found that the presence of hypoperfusion in parietal, temporal, and frontal lobes was 56.3% in PSS cases with neuropsychiatric symptoms and findings and 17.6% in PSS cases with no neuropsychiatric symptoms and findings at the brain Tc‐ECD SPECT and this result increased the sensitivity of the brain Tc‐ECD SPECT (Ibn Yacoub et al., 2012). It was revealed that in PSS cases, there was a correlation between cognitive dysfunction and decreased NAA level and decreased NAA/Cr rate at the subcortical frontal and basal ganglia's white matter and presence of the increased vascular resistance in MCA circulation and this was explained by the subclinical vascular inflammation and endotheliitis in PSS (Morreale et al., 2014). These results support the fact that subcortical cognitive function disorders may be due to organic reasons and is explained by the vasculopathy based on small vein vasculitides in pathogenesis of the SS (Gerraty et al., 1993; Le Guern et al., 2010; Mataro et al., 2003).

It was revealed that there was an increase in the presence of subjective cognitive symptoms, frequency of depression, and fatigue severity in PSS compared to healthy control (Martinez et al., 2010; Mataro et al., 2003; Morreale et al., 2014; Segal et al., 2012). A strong correlation was observed between the presence of subjective cognitive symptoms and depression, fatigue severity and pain severity, quality of life, and dysfunction in attention, executive functions, working memory, and verbal memory among objective cognition tests (Coates et al., 1999). In the series of Segal et al. (2012), the subjective cognitive symptoms, depression, fatigue severity, and pain scores were found to be higher compared to the healthy control group. In the PSS patient group, prevalence of depression was 47% and the presence of subjective symptoms was more in the depressed cases. PSS cases with depression had a low performance only in Wisconsin Card Sorting Test (WCST) measuring the executive functions in a statistically significant way compared to cases without depression and also had a low performance in Trails Making Test B evaluating attention, working memory, and executive functions and in HVLT test evaluating verbal memory compared to cases without depression even though it did not show a statistically difference. In PSS group, there was a positive correlation between the subjective symptoms, depression score, and fatigue severity, and also a positive correlation was found between the severity of the subjective cognitive symptom and low verbal memory (*p *= .048). The objective determination of depression and pain existence affected negatively attention and working memory in the cognitive tests (Segal et al., 2012). It is known that depression and pain decrease working memory and executive function's performance; however, verbal memory is not affected by pain and depression (Lafitte et al., 2001), therefore patients with depression were excluded from the study. In this study no statistically significant difference was observed in terms of all neuropsychological tests, depression, fatigue severity, health state, and daily‐life activities in between PSS and control cases without depression. These findings indicate the presence and pattern of cognitive impairment without the negative effects of depression in patients with PSS.

Some studies revealed that the cognitive function disorder identified in PSS can recover over time with corticosteroid or immunosuppressive treatment or in the period without treatment (Martinez et al., 2010; Yoshikawa et al., 2012). In 20 case series of Martinez et al. (2010), it was found that at basal and the neuropsychological evaluation, 8 years later verbal and visual memory had dysfunction in simple reaction time, Stroop, Trail Making Test B, and RCFT tests, but increasing scores were obtained at visual and verbal memory, WCST, and CPT reaction time. This situation may support that there is no progressive dementia in PSS (Martinez et al., 2010; Yoshikawa et al., 2012). In the depression and fatigue scores, there was no change at the end of 8 years follow‐up (Martinez et al., 2010). Therefore, administration of detailed neuropsychological tests is useful in the diagnosis of subclinical cognitive disorder and for directing the treatment.

In recent years, there is a limited number of studies that examine the effect of the disease on quality of life in PSS. Decreased quality of life has already been revealed in patients with PSS by using the Short‐Form 36. It were showed that all eight scales of the SF‐36 significantly decreased in patients with PSS (Baturone et al., 2009; Champey et al., 2006; Ibn Yacoub et al., 2012; Inal, Kitapcioglu, Karabulut, Keser, & Kabasakal, 2010; Lendrem et al., 2014; Meijer et al., 2009; Segal et al., 2009; Strömbeck et al., 2003). In 57 case series of Ibn Yacoub et al. (2012), it was found that compared to general population, lower performance was found in all SF‐36 scores, including physical problems, role functioning emotional, vitality, and general health scales. In the study of Baturone et al. (2009), 30 PSS cases had mean scores of <50 in each of the eight scales of SF‐36 compared to 20 healthy normal individuals. In the study of Champey et al. (2006), 109 PSS cases had lower scores in each of eight scales compared to 443 normal female individuals. In our study, we found decreased quality of life in patients with PSS according to EQ‐5D1, EQ‐5D2, and the Short‐Form 36, but there were no statistical difference in scores of physical function, social functioning, and mental health of the Short‐Form 36 in PSS patients. It shows that physical and social function and psychological distress and well‐being of PSS are not significantly affected. Patients with PSS had increased serum levels of Th1 (IL‐1, IL‐2, srIL‐2), Th2 (IL‐6, IL‐10) cytokines, TNF‐alpha, and IFN‐gamma when compared to healthy controls (Baturone et al., 2009; Garcíc‐Carrasco et al., 2001). Baturone et al. (2009) showed that there was a significantly negative correlation between PCS of the SF‐36 and only serum levels of IL‐6 in PSS patients, and the PSS patients with the higher concentration of IL‐6 showed a significantly lower value in PCS than those with a lower concentration. This study supports adverse effect of decreased anti‐inflammatory cytokines on quality of life in PSS cases.

It is shown that fatigue and depression are the variables affecting the quality of life that is measured by SF‐36 (Champey et al., 2006; Meijer et al., 2009; Segal et al., 2009). Depression is reported as a factor related to a decreased quality of life in patients with PSS (Ibn Yacoub et al., 2012). Champey et al. (2006) stated that the strong correlation between PCS and MCS of the SF‐36 and psychological distress level in PSS patients emphasized the importance of the psychological dimension in results of the SF‐36. Segal et al. (2009) showed that somatic fatigue was the main predictor of physical function and the general health, and on the other hand, depression was the key predictor of emotional well‐being. Meijer et al. (2009) revealed that fatigue was independently associated with a reduced physical component score and mental component score of SF‐36. EQ‐5D questionnaire is another test evaluating the quality of life in PSS cases. EQ‐5D utility values combine physical and psychological well‐being in scores (The EuroQol Group, 1990). Lendrem et al. (2014) showed that the patients with PSS reporting any problem in mobility, self‐care, usual activities, pain/discomfort, and anxiety/depression were 42.2%, 16.7%, 56.6%, 80.6%, and 49.4%, respectively, compared to 5.4%, 1.6%, 7.9%, 30.2%, and 15.7%, respectively, in general population. They mentioned depression and pain as the most important predictors of EQ‐5D utility values, and fatigue, anxiety, and body mass index were other statistically significant predictors of utility values in lesser variability in PSS. The weak effect of somatic fatigue on EQ‐5D is explained by the combination of physical and psychological component of EQ‐5D.

Fatigue is the most common symptom in patients with PSS. Abnormal fatigue is defined as enduring and generalized tiredness and can be characterized in terms of intensity, duration, and effects on daily activities (Segal et al., 2008). A number of studies revealed that there is a high prevalence of fatigue in PSS patients between 67% and 87.5% (Barendregt et al., 1998; Bax, Vriesendorp, Kallenberg, & Kalk, 2002; Bowman, 2008; Giles & Isenberg, 2000; Haldorsen, Bjelland, Bolstad, Jonsson, & Brun, 2011; Harboe et al., 2009; Hartkamp et al., 2004; Lwin, Bishay, Platts, Booth, & Bowman, 2003; Priori et al., 2010; Segal et al., 2008; Strömbeck, Theander, & Jacobsson, 2005; Wouters et al., 2012). Previous studies indicated that patients with PSS had more severe symptoms of fatigue than controls on all subscales of fatigue such as degree of fatigue, reduced activity, and its effect on daily living (Harboe et al., 2009; Hartkamp et al., 2004; Ibn Yacoub et al., 2012). In the focusing on correlation between VAS scores for fatigue and both PCS of quality of life and psychological distress in PSS patients, PCS of quality of life and psychological distress are closely affected from fatigue (Champey et al., 2006). In addition, Ibn Yacoub et al. (2012) showed that there was a high negative correlation between VAS fatigue scores, fatigue severity, and physical and psychological components of SF‐36. Depression was reported more frequently in patients with PSS than controls, and the prevalence of depression was reported to be 32–45.8% (Bowman, 2008; Harboe et al., 2009; Lafitte et al., 2001; Morreale et al., 2014; Segal et al., 2008). Recent studies have showed a significant correlation between the severity of fatigue and the level of depression, and depression has been considered as a strongest predictor of fatigue (Barendregt et al., 1998; Bax et al., 2002; Segal et al., 2008). Segal et al. determined significantly higher mean FSS scores in the group of subjects with depression (mean FSS score 5.5 ± 1.3 in depressed patients and 4.2 ± 1.5 in nondepressed patients, p .001), but 59% fatigued patients were not depressed and they reported that depression is not the primary cause of fatigue in PSS (The EuroQol Group, 1990). We found that all the depression, fatigue severity, health state, and quality of life tests showed a significant positive correlation with each other.

In conclusion, using the comprehensive neuropsychological tests to assess the cognitive performance can determine the subclinical and clinical cognitive dysfunction in the patients of PSS. Clock Drawing, PASAT, and AVLT are very useful tests to determine attention, information processing speed, executive functions, and short‐term and long‐term verbal memory in the patients with PSS. Depression and fatigue may not affect the neuropsychological tests performance in well‐selected patients. All the depression, fatigue severity, health state, and quality of life tests showed a significant positive correlation with each other.

## Conflicts of Interest

None declared.
